# Overactive bladder symptom severity, bother, help-seeking behavior, and quality of life in patients with type 2 diabetes: a path analysis

**DOI:** 10.1186/s12955-017-0829-z

**Published:** 2018-01-02

**Authors:** Dongjuan Xu, Meng Zhao, Liqun Huang, Kefang Wang

**Affiliations:** 1 School of Nursing, Shandong University, No. 44, Wenhua Xi Road, Jinan, Shandong 250012 People’s Republic of China; 2 School of Nursing, Purdue University, West Lafayette, Indiana 47907 USA

**Keywords:** Overactive bladder, Symptom severity, Quality of life, Help-seeking behavior, Bother

## Abstract

**Background:**

This study aimed to investigate the relationships among overactive bladder (OAB) symptom severity, bother, help-seeking behavior, and quality of life (QOL) in patients with type 2 diabetes.

**Methods:**

A total of 127 diabetic patients, aged at least 18 years, with overactive bladder from a hospital in Shandong Province, China, were recruited for this study. Symptom severity, bother, and quality of life were assessed using the Overactive Bladder Symptom Score (OABSS), Patient Perception of Bladder Condition (PPBC), and Overactive Bladder Questionnaire Short Form (OAB-q SF), respectively. Help-seeking behavior was assessed by asking patients whether they consulted health care professionals or received treatment for their bladder problems. A two-step path analysis was performed to analyze the data.

**Results:**

OAB symptom severity was directly associated with lower levels of QOL, and the strength of this association was no longer significant when taking bother and help-seeking behavior into account. Bother increased with OAB symptom severity, and patients with bothersome OAB tended to have lower levels of QOL. Moreover, bother increased help-seeking behavior; however, patients who sought help tended to have lower levels of QOL.

**Conclusions:**

Our findings highlight the role of bother and help-seeking behavior in the relationship between OAB symptom severity and QOL. To improve a patient’s QOL, health care providers should focus not only on symptom bother but also on dysfunctional help-seeking patterns.

## Background

Overactive bladder (OAB) syndrome is defined as “urinary urgency, usually accompanied by frequency and nocturia, with or without urgency urinary incontinence” [1]. Nearly 11% of the worldwide population (455 million) is affected by OAB, and the burden is expected to be the greatest in Asia [2]. In China, OAB symptoms have been found to affect more than 1 in 5 adults aged ≥40 years [3]. The total cost is estimated to be at $65.9 billion in 2007 in the Unites States alone [4]. The financial cost is substantial not only in Western countries [5], but also in Asian countries [6]. Moreover, previous literature consistently presented a negative association between OAB and QOL, using either a generic or a disease-specific QOL measure [7, 8].

With respect to the factors influencing QOL in patients with OAB, empirical studies have emphasized on OAB symptom severity as a key determinant [9, 10]. Vaughan et al. found that increased OAB symptom severity was directly associated with QOL deterioration across all dimensions [9]. In addition, a positive association between OAB symptom severity and experience of symptom bother was noted [10, 11]. It was not only urge incontinence that patients found debilitating; nocturia and urgency were also considered bothersome [12, 13]. However, symptom severity did not engender bother straightforwardly [14, 15]. Some individuals were severely bothered by a small amount of leakage, whereas others were only slightly bothered by heavy leakage [15]. A previous study found that roughly one in three with urge incontinence and one in seven with urgency reported moderate or major bother [9]. Moreover, the experience of symptom of bother adversely affected many aspects of QOL, including daily life activities, work productivity, social interactions, and emotional well-being [16]. Therefore, investigating the relationships among OAB symptom severity, bother, and QOL is essential.

Although only a small proportion of affected individuals actually consulted physicians or received treatment for OAB [8, 17], previous studies have indicated that perceived symptom severity and degree of bother are key reasons for help-seeking behavior [13, 14]. In addition to a direct relationship, symptom severity also had an indirect relationship with help-seeking behavior, i.e. via bother [18]. Bother appears to influence help-seeking behavior more than the symptoms themselves do [19]. Irwin et al. showed that about 52% of bothered individuals with OAB reported consulting their physicians, whereas 21% were not bothered [12]. Xu et al. also found that only women bothered by incontinence had intentions to seek help, regardless of symptom severity [15]. Individuals with OAB benefit from consulting their physicians and receiving treatments for their symptoms, especially as effective treatments are available. Behavioral interventions, such as pelvic floor muscle training, bladder training, and urge suppression techniques, can be used to extend voiding interval and reduce urgency and incontinence [20–22]. Moreover, behavioral interventions may increase the efficacy of pharmacotherapy [22], which consequently helps alleviate symptoms, prevent OAB recurrence, and improve QOL. However, previous studies have not considered OAB symptom severity, bother, help-seeking behavior, and QOL together. Thus, little is known about the role of bother and help-seeking behavior in the relationship between symptom severity and QOL.

**Fig. 1:**
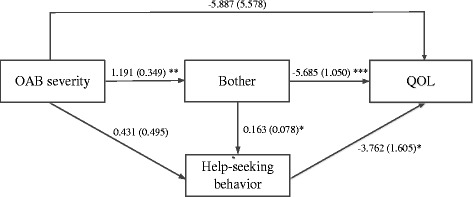
Path diagram of the relationships among OAB severity, bother, help-seeking behavior, and QOL. Path coefficients with standard errors in parentheses are shown along the path arrows. The path analysis was adjusted for age, sex, years of education, marital status, living area, income, body mass index, urinary tract infection history, Charlson Comorbidity Index, duration of diabetes, and diabetes treatment (insulin or anti-diabetic medication) *p < 0.05, **p < 0.01, ***p < 0.001.

This study was conducted using the existing data from OAB patients with type 2 diabetes. Diabetes contributes to an earlier onset and increases severity of bladder dysfunction [23], and OAB occurs more commonly in patients with type 2 diabetes than in the general adult population [24]. Thus, this study aimed to investigate the relationships among OAB symptom severity, bother, help-seeking behavior, and QOL in patients with type 2 diabetes in Mainland China, as illustrated in Fig. 1. Our hypotheses are as follows:

- Hypothesis 1. OAB symptom severity is associated with lower levels of QOL.
- Hypothesis 2. Bother increases with OAB symptom severity, and patients with bothersome OAB are more likely to have lower levels of QOL.
- Hypothesis 3. Both OAB symptom severity and bother are expected to be associated with help-seeking behavior, and patients who sought help are more likely to have higher levels of QOL.
- Hypothesis 4. The strength of the association between OAB symptom severity and QOL assumed in hypothesis 1 is attenuated when taking bother and help-seeking behavior into account.

## Methods

### Study design and sample

This is a cross-sectional design. This study included a subpopulation of a larger study measuring OAB, toileting behaviors, and QOL among patients with type 2 diabetes [25]. The diabetic patients were recruited from May to August 2014 from the endocrinology outpatient department of a non-profit teaching hospital, which is one of the two largest hospitals in Shandong Province, China. The larger study had 1025 eligible diabetic patients, of which 142 patients had OAB. Fifteen patients with OAB had missing data and thus were excluded from the analyses. Consequently, a total of 127 diabetic patients with OAB were included in this study. The inclusion criteria were as follows: (1) ≥18 years old, (2) diagnosed as having type 2 diabetes, (3) urgency score of Overactive Bladder Symptom Score ≥ 2 and a total score ≥ 3, and (4) willing to participate in the study and capable of understanding study procedure and questions. The exclusion criteria were the following: (1) neurological disorders (such as stroke, Parkinson’s disease, and multiple sclerosis), (2) active urinary tract infections, (3) history of bladder surgery, and (4) pelvic organ prolapse.

This study was approved by the Institutional Review Board of Shandong University. Written informed consent was obtained from each patient before the survey. The survey was completed anonymously, and the patients were assured that their responses would be kept confidential.

### Measures

#### Participant characteristics

Health-related patient characteristics included body mass index (BMI), urinary tract infection history, comorbidity, duration of diabetes, and diabetes treatment (insulin and/or anti-diabetic medication). The Charlson Comorbidity Index (CCI) was used to measure a range of comorbid conditions for each patient. In this study, all patients had diabetes; thus, diabetes was not included in the calculation of CCI [26]. Each condition had a corresponding weight (1, 2, 3, or 6), depending on mortality risk. A single comorbidity score was calculated for each patient by adding all the weights. The higher the score, the more likely mortality is predicted.

#### OAB symptom severity

The Overactive Bladder Symptom Score (OABSS) was used to quantify OAB symptoms. The questionnaire was originally developed by Homma et al. [27], and was later validated by the Taiwanese Continence Society in the Chinese population [28]. The test-retest reliability of the Chinese OABSS was moderate to good [28] and the Cronbach’s alpha coefficient was 0.65 in this study. The total OABSS is the sum of four symptom scores: daytime frequency (score 0–2), nighttime frequency (score 0–3), urgency (score 0–5), and urgency incontinence (score 0–5) [27]. The total score ranges from 0 to 15, with a higher score indicating more severe OAB symptoms. In this study, all participants had an urgency score ≥ 2 and a total score ≥ 3. In the analyses, we dichotomized OAB symptom severity into minor (total score ≤ 5) and serious (total score > 5) OAB.

#### OAB bother

The Patient Perception of Bladder Condition (PPBC) questionnaire, a single-item, 6-point scale (1 = “no problems at all” to 6 = “many severe problems”), was used to assess the OAB patients’ impression of their bladder problems [29]. Construct validity, responsiveness, and test-retest reliability have been well-validated [29].

#### Help-seeking behavior

Help-seeking behavior was assessed by asking patients the following question: “Have you ever consulted health care professionals or received treatment for your urinary symptoms?”

#### OAB-specific QOL

The Overactive Bladder Questionnaire Short Form (OAB-q SF) was used to assess the effect of OAB symptoms on health-related quality of life [30]. The instrument has good internal consistency reliability, test-retest reliability, construct validity, and responsiveness [30]. The Cronbach’s alpha coefficient was 0.72 in this study. The OAB-q SF consists of three QOL domains: coping, sleep, and emotional/social interaction. All scale scores were transformed to a 0- to 100-point scale, with lower scores indicating greater effect, i.e., worse QOL.

### Statistical analysis

We used descriptive statistics to describe the sociodemographic and health-related characteristics of diabetic patients with OAB. To test our hypotheses on the relationships among OAB symptom severity, bother, help-seeking behavior, and QOL, as illustrated in Fig. 1, we conducted a two-step path analysis. Specifically, we used path analysis because it has the ability to test hypothesized relationships of a complex model. First, we explored the direct relationship between OAB symptom severity and QOL, without considering bother and help-seeking behavior in the model. If there was a significant relationship between OAB symptom severity and QOL, we then tested the full model by adding bother and help-seeking behavior in the path analysis. All models were adjusted for age, sex, education, marital status, living area, income, BMI, urinary tract infection history, CCI, duration of diabetes, and diabetes treatment. Only two patients had a different race/ethnicity; thus, we did not control race/ethnicity in the path analysis. The goodness of model fit was evaluated using comparative fit index (CFI), Tucker-Lewis index (TLI), and root mean square error of approximation (RMSEA). A CFI and TLI > 0.90 and a RMSEA < 0.06 indicate a good fit to the data [31]. All statistical analyses were performed using Mplus (version 7.31; Muthén & Muthén, Los Angeles, CA). Statistical significance was accepted at the *p* < 0.05.

## Results

Table 1 shows the characteristics of 127 diabetic patients with OAB. The mean age was approximately 63 years, and 46% of diabetic patients with OAB were women. The average duration of diabetes was 10.31 years, and 91.34% of patients received insulin therapy or anti-diabetic medication. Thirty-five patients (27.56%) had minor OAB and 92 (72.44%) had serious OAB. With regard to bother, 14.17% reported that their bladder condition caused them severe problems, and 7.87% reported bladder condition caused them many severe problems. However, only 22.05% had a previous help-seeking behavior for their bladder condition. The score for OAB-specific QOL was about 72.76 (range 0–100).

**Table 1:** Characteristics of diabetic patients with OAB (*N* = 127)

Variables	Mean ± SD or *N* (%)
Age (years)	63.16 ± 11.17
Race/ethnicity	
Han	125 (98.43%)
Other	2 (1.57%)
Sex	
Female	59 (46.46%)
Male	68 (53.54%)
Years of education	9.07 ± 4.63
Marital status	
Married	107 (84.25%)
Single/divorced/widowed	20 (15.75%)
Living area	
Urban	100 (78.74%)
Rural	27 (21.26%)
Income (RMB (USD)/month)	
≤ 3000 (451)	86 (67.72%)
> 3000 (451)	41 (32.28%)
Body mass index (BMI)	25.59 ± 3.87
Urinary tract infection history^a^	
Yes	11 (8.66%)
No	116 (91.34%)
Charlson Comorbidity Index (CCI)^b^	1.98 ± 1.16
Duration of diabetes (years)	10.31 ± 8.31
Receiving diabetes treatment	
Yes	116 (91.34%)
No	11 (8.66%)
OAB severity	
Serious	92 (72.44%)
Minor	35 (27.56%)
OAB bother	3.06 ± 1.59
Previous help-seeking behavior	
Yes	28 (22.05%)
No	99 (77.95%)
Quality of life	72.76 ± 20.61

*SD* standard deviation, *OAB* overactive bladder, *RMB* Chinese Yuan

^a^Patients who had a urinary tract infection at least 1 month before the survey

^b^Diabetes was not included in the calculation of Charlson Comorbidity Index (CCI)

The path analysis revealed the relationships among OAB symptom severity, bother, help-seeking behavior, and QOL (Table 2 and Fig. 1). A direct relationship between OAB symptom severity and QOL was found, indicating that the more severe the OAB symptom, the lower the QOL (model 1 in Table 2). However, when bother and help-seeking behavior was added in the model, the direct relationship between OAB symptom severity and QOL was attenuated and no longer significant (model 2 in Table 2). However the indirect relationship was significant: OAB symptom severity was positively associated with bother, which in turn had a negative association with QOL (Fig. 1). Moreover, bother had a positive association with help-seeking behavior, which in turn had an unexpected negative association with QOL. No direct relationship between OAB symptom severity and help-seeking behavior was observed. Thus, the more severe the OAB symptom, the more likely the patients felt bothered; patients bothered by their bladder problems tended to have a lower QOL and were more likely to seek help. Contrary to expectations, help-seeking behavior decreased QOL. The model indexes (RMSEA, CFI, and TLI) indicated that the full model (model 2) fitted the data well.

**Table 2:** The relationships among OAB severity, bother, help-seeking behavior, and QOL

	Model 1 without bother and previous help-seeking behavior			Model 2 with bother and previous help-seeking behavior		
	Coefficient	SE	*P* value	Coefficient	SE	*P* value
OAB severity	−15.139	3.556	<0.001	−5.887	5.578	0.291
Bother	–	–	–	−5.685	1.050	<0.001
Previous help-seeking behavior	–	–	–	−3.762	1.605	0.019
Age	−0.117	0.185	0.528	−0.117	0.203	0.565
Sex (female vs. male)	1.299	3.320	0.696	1.295	3.778	0.732
Years of education	−0.176	0.398	0.659	−0.176	0.458	0.701
Marriage (married vs. unmarried)	2.821	4.438	0.525	2.737	4.726	0.562
Living area (urban vs. rural)	3.208	4.748	0.499	3.318	5.280	0.530
Income (>3000 vs. ≤3000)	5.270	3.571	0.140	5.281	4.243	0.213
Body mass index (BMI)	−0.375	0.403	0.352	−0.376	0.414	0.364
Urinary tract infection (UTI)^a^	−13.020	5.576	0.020	−13.034	5.868	0.026
Charlson Comorbidity Index (CCI)^b^	−3.621	1.539	0.019	−3.621	1.722	0.035
Duration of diabetes	−0.511	0.240	0.033	−0.511	0.237	0.031
Receiving diabetes treatment	14.463	5.779	0.012	14.507	7.163	0.043

*OAB* overactive bladder, *QOL* quality of life, *SE* standard error, *RMB* Chinese Yuan

^a^Patients who had a urinary tract infection at least 1 month before the survey

^b^Diabetes was not included in the calculation of Charlson Comorbidity Index (CCI)

## Discussion

To the best of our knowledge, this is the first quantitative study to investigate the relationships among OAB symptom severity, bother, help-seeking behavior, and QOL in patients with type 2 diabetes using a path analysis. Our findings supported most of our hypotheses. OAB symptom severity was directly associated with lower levels of QOL (hypothesis 1), and the strength of this association was no longer significant when taking bother and help-seeking behavior into account (hypothesis 4). Bother increased with OAB symptom severity, and patients with bothersome OAB tended to have lower levels of QOL (hypothesis 2). Hypothesis 3 was partially supported as only bother was related to help-seeking behavior. In contrast to our expectations, patients who sought help tended to have lower levels of QOL. Our findings provide important implications for the management of OAB in patients with diabetes to improve their QOL, with particular regards to help-seeking behavior.

The direct association between OAB symptom severity and lower levels of QOL is consistent with the findings of previous studies [9, 10, 32]. We further demonstrated that this direct association was attenuated and no longer significant after considering bother and help-seeking behavior. This result is somehow related to a previous finding indicating that gastrointestinal symptom severity is not only directly but also indirectly associated with QOL, which is mediated by bother (i.e., psychological distress) [33]. The major difference between previous studies and our study is the consideration of both bother and help-seeking behavior; other researchers have only looked into bother. The different study population may have also contributed to the dissimilar results.

Similar to earlier reports [10, 11, 15, 34], we found a positive association between perceived OAB symptom severity and degree of bother and pointed at the central role of bother, which was a primary trigger for help-seeking behavior and related to lower QOL. The higher the degree of bother, the more likely an individual seeks for help [13, 14, 35]. A number of studies indicated that individuals with OAB were bothered by the need to use absorbent products, limited clothing choices, mapping out toilet locations, frequent voiding to prevent leakage episodes, concerns about urine odor, or self-imposed lifestyle constraints [36, 37]. These negative feelings would disrupt their daily lives and impair their well-being, supporting previous findings that OAB has a detrimental effect on QOL.

The beneficial effect of help-seeking behavior on QOL was not supported by this study. Our results indicated that help-seeking behavior was negatively associated with QOL. Some studies identified misconceptions and miscommunication between patients and providers, and patients’ dissatisfaction with the care they had received [38, 39].For example, patients had unrealistic expectations that their OAB problems could be cured with medication alone, and side effects, which were seldom understood by patients, often resulted in discontinuation [38, 39]. In addition, some physicians failed to discuss the importance of behavioral modification with patients [38]. Botelho et al. stated that some health care providers have possibly assured that OAB symptoms are “normal,” which could influence future help-seeking behavior [14]. Moreover, health care providers often attribute bladder symptoms to diabetes-related polyuria. Because of the misconceptions, miscommunication, and poor compliance with treatment, help-seeking behavior negatively affects QOL.

### Limitations

Our study has some limitations. First, the small sample size and selection of diabetic patients from one hospital constrained the generalizability of the findings. Second, the causal inferences regarding the direction of the relationships illustrated in Fig. 1 are limited because of the cross-sectional nature of this study. According to the cognitive behavioral theory [40], a stimulus or trigger (OAB symptom severity) activates cognitive processes (bother), which in turn influence behavioral and emotional aspects (help-seeking behavior and QOL). It is also possible that poor QOL may worsen a patient’s feeling of bother and lead to help-seeking behavior for OAB. Longitudinal studies to fully understand the direction of the relationships among symptom severity, bother, help-seeking behavior, and QOL are warranted. Third, satisfaction, knowledge, and experience after receiving treatment or consultation from health care providers were not measured; thus, we were not able to assess the underlying reasons for the association between help-seeking behavior and worse QOL.

## Conclusions

This study addressed a gap in the literature by examining the role of bother and help-seeking behavior in the relationship between OAB symptom severity and QOL among patients with type 2 diabetes. To improve patients’ QOL, health care providers should focus not only on symptom bother but also on dysfunctional help-seeking patterns. Nearly 80% of individuals with OAB had never sought help for their urinary symptoms, despite the problem being treatable or at least manageable. Moreover, this study’s findings highlight the need for health education and promotion to improve patients’ help-seeking behavior and health care provider’s management of OAB symptoms during diabetic care. Furthermore, help-seekers’ relatively poor QOL may be due to misconceptions and miscommunication with health care providers or poor compliance with treatment. Thus, promoting effective provider-patient communication, thereby increasing patients’ understanding of bladder issues and adherence to treatments, is crucial to achieve satisfaction and improved QOL.

## Abbreviations

CCI: Charlson Comorbidity Index
CFI: Comparative fit index
OAB: Overactive bladder
QOL: Quality of life
RMSEA: Root mean square error of approximation
TLI: Tucker-Lewis index
